# Dysregulation of HULC promotes contrast-induced nephropathy (CIN) via regulating signaling pathway of miRNA-512 and prostaglandin E1 (PGE_1_)

**DOI:** 10.1038/s41598-020-68634-7

**Published:** 2020-07-16

**Authors:** Liang Zhang, Pan Li, Bi-li Zhang, Man-li Yu, Rong-liang Xu, Hong Wu, Shao-ping Chen

**Affiliations:** Department of Cardiology, The First Affiliated Hospital of the Second Military Medical University, No. 168 Changhai Road, Yangpu District, Shanghai, People’s Republic of China

**Keywords:** miRNAs, Kidney

## Abstract

It has been shown that contrast-induced nephropathy (CIN) can be attenuated by the administration of PGE1. As an enzyme responsible for the production of PGE1, PTGS1 was confirmed in this study as a miR-512 target. Meanwhile, HULC has been identified as a competing endogenous RNA of miR-512. Therefore, in this study, we tested the diagnostic value of HULC and miR-512 in subjects with or without CIN. In addition, we evaluated the regulatory relationship among HULC, miR-512, PTGS1 and PGE1 in vitro. We enrolled 320 patients with coronary heart disease and divided them into a CIN group and a non-CIN group. Subsequently, we detected the differential expression of miR-512, HULC and PGE1 in the two groups. We also used a dual luciferase reporter assay to evaluate the regulatory relationship among HULC, miR-512, PTGS1 and PGE1 in THP-1 cells. In patients with CIN, the expression levels of HULC and PGE1 were lower, but the expression level of miR-512 was higher. MiR-512 could directly bind to and negatively regulate the expression of PTGS1 and HULC. The expression of HULC was positively correlated with the expression of PTGS1 and PGE1, while negatively correlated with the expression of miR-512. The findings of this study demonstrated that deregulation of lncRNA-HULC/miR-512/PTGS1/PGE1 might be involved in the pathogenesis of CIN.

## Introduction

As a form of kidney damage induced by recent contrast medical imaging exposure without other significant reasons for acute kidney injury, contrast-induced nephropathy (CIN) represents a disorder featured by a rise in serum creatinine (Scr) > 25% from baseline and/or an absolute increased Scr from baseline of > 0.5 mg/dL, which usually occurs after the use of a contrast media^1^. CIN can cause high mortality and morbidity, especially in patients undergoing percutaneous coronary intervention (PCI) or coronary angiography. There is no effective way to prevent CIN, and hence many researchers tried to develop new pharmacological agents with the potential to reduce the risk of CIN^2^.

Miao et al. found that prostaglandin E1 (PGE1) inhibits the development of CIN in patients receiving contrast computed tomography^3^. Another study also showed that the oral administration of pentoxifylline can prevent CIN to a certain degree^4^. In addition, as a widely used prophylactic agent in patients receiving computed tomography, N-acetylcysteine reduces CIN risk^5^. In many cancers such as bladder cancer, the level of cyclooxygenase-2 (COX-2), an enzyme participating in the biosynthesis of prostaglandin E2 (PGE2), is elevated^6^. In a rat model of bladder cancer triggered by *N*-butyl-4-*N*-(4-hydroxybutyl) nitrosamine, the level of COX-2 is also shown to be increased along with higher expression of PGE2^7,8^. In humans, the expression of COX-2 can be easily detected in glomerular podocytes, while the patients suffering from acute rejection of renal allograft show up-regulated expression of PGE2^9,10^. Similarly, it was shown that the number of COX-2-expressing podocytes, macula densa cells and mesangial cells was increased in adult rats of diabetes, whereas the suppression of COX-2 reduced the severity of proteinuria and delayed the progression of diabetic nephropathy^11^.

Small RNA molecules do not code for proteins, and microRNAs (miRNAs), one of the small RNA molecules, were reported to mediate gene expression post-transcriptionally by targeting the 3′ UTRs of target mRNAs^12^. Moreover, long-noncoding RNAs (lncRNAs) have a length of greater than 200 nt and can also regulate gene expression. Recently, many studies demonstrate that lncRNAs and miRNAs are involved in cell growth and differentiation, whereas the interactions between lncRNAs and microRNAs may be implicated in many diseases^13,14^.

It has been shown that CIN could be attenuated by the administration of PGE1^15,16^. As an enzyme responsible for the production of PGE1, PTGS1 was confirmed in this study as a target of miR-512. Meanwhile, we also found that HULC has been reported to be a competing endogenous RNA (ceRNA) to sponge miR-512^17–19^. Therefore, in this study, we tested the diagnostic value of HULC and miR-512 in subjects with or without CIN. In addition, we evaluated the regulatory relationship among HULC, miR-512, PTGS1 and PGE1 in vitro.

## Materials and methods

### Patient grouping

In this study, we enrolled 320 subjects of coronary heart diseases. All patients were treated by coronary intervention, including 78 CIN subjects (the CIN group) and 242 patients free of CIN (non-CIN group). All subjected enrolled in this study were diagnosed with coronary heart disease and have received angiogram. Those who were diagnosed with CIN (CIN are defined as a rise in SCr > 25% from baseline, or by an absolute increase from baseline of > 0.5 mg/dL) were divided into CIN group and those who are free with CIN were divided into non-CIN group. In addition, those who were diagnosed with any renal disease other than CIN or have been currently or recently (within past 3 months) diagnosed with any infection, inflammation or immune diseases were excluded from this study.

Peripheral blood samples were collected and HULC and miR-512 expression were detected in these two groups. This study was approved by the Ethics Committee of the First Affiliated Hospital of the Second Military Medical University and was performed in accordance with the latest vision of the Declaration of Helsinki. All patients signed the form of informed consent before the study was initialized.

### RNA isolation and real-time PCR

Total RNA from cultured cells and peripheral blood samples was extracted using Trizol. The concentration of extracted RNA was then measured by spectrometer. Subsequently, a TaqMan Reverse Transcription Kit (Applied Biosystems, Foster City, CA) was adopted to convert total RNA into cDNA, followed by real-time PCR on an ABI Prism 7,500 system (ABI, Foster City, CA) using a SYBR green mix following the kit instruction. The relative expression of HULC, miR-512, and PTGS1 mRNA was calculated using the 2^−ΔΔCT^ method with GAPDH (for lncRNA and mRNA) and U6 (for miRNAs) being utilized as the internal control.

### Cell culture and transfection

THP-1 cells were maintained in DMEM containing 10% FBS, 4.5 g/L glucose, and 100 units/mL of streptomycin-penicillin. The cells were incubated at 37 °C in an atmosphere of 5% CO_2_. Subsequently, the cells were transfected with the control or HULC siRNA at the concentration of 50 nM, and an empty vector or pcDNA-HULC using Lipofectamine 2000 (Invitrogen, Carlsbad, CA) at the concentration of 200 ng/L, followed by the measurement of target gene expression at 48 h post transfection.

### Vector construction, mutagenesis and luciferase assay

PCR was used to amplify the full length of HULC and 3ʹ-UTR PTGS1 mRNA containing the binding site for miR-512, and the PCR products were inserted into pcDNA vectors (Promega, Madison, WI) to obtain the plasmids for the wild-type HULC and PTGS1 3ʹ-UTR. Subsequently, a QuickChange Mutagenesis kit (Stratagene, La Jolla, CA) was adopted to carry out site-directed mutagenesis in the miR-512 binding site of HULC and PTGS1 mRNA, and the mutagenesis products were also inserted into pcDNA vectors to obtain the plasmids for the mutant HULC and PTGS1 3ʹ-UTR. Subsequently, THP-1 cells were co-transfected with the mutant-type or wild-type HULC/PTGS1 3′-UTR, in conjunction with miR-512 mimics or a negative control (NC), using Lipofectamine 2000 (Invitrogen, Carlsbad, CA). During transfection, a Renilla luciferase reporter construct (pRL-TK) was used as the internal standard. Finally, the transfected THP-1 cells were collected 48 h later and the luciferase activity in the cells was measured on a Berthold AutoLumat LB9507 rack luminometer (Berthold Technologies, Shanghai, China) using a Dual Luciferase Assay (Promega, Madison, WI).

### Western blot analysis

Peripheral blood samples and transfected THP-1 cells were lysed and the proteins in the supernatant were collected by centrifugation. Then, the isolated proteins were resolved by 10% SDS-PAGE and blotted onto a PVDF membrane. Subsequently, the membrane was blocked by 5% non-fat milk for 1 h at 37 °C and then incubated at 4 °C overnight with anti-PTGS1, anti‑ATG7, anti‑LC3‑II and anti-β-actin (Internal control) primary antibodies (Santa Cruz Biotechnology, Santa Cruz, CA), followed by 1 h of room temperature incubation with HPR-conjugated secondary antibodies (Abcam, Cambridge, MA). Then, an ECL kit (GE Healthcare, Little Chalfont, UK) was used to develop the membrane and the target bands were quantified using Image J (NIH Image, Bethesda, MD) .

### Cell apoptosis analysis

The apoptosis of THP-1 was detected by flow cytometry using an Annexin V FITC‑conjugated/propidium iodide (PI) apoptosis kit (Nanjing Keygen Biotech Co., Ltd., Nanjing, China). Different groups of THP-1 cells were seeded into a 24-well plate at a density of 1 × 10^5^ cells/well and were incubated with serum-free medium for 12 h after the cell confluence reached 70–80%. The incubated cells were subsequently collected and incubated with 5 μl Annexin V‑FITC and 5 μl PI for 30 min at room temperature before being analyzed with a flow cytometer (BD Biosciences, San Jose, CA, USA).

### Statistical analysis

All results were presented as mean ± standard deviations. ANOVA was employed to compare the results among different groups. ROC curves were plotted and analyzed using Chi-square test and Pearson correlation analysis. All analyses were conducted in SPSS 21.0 (IBM, Chicago, IL). *P* < 0.05 was considered significant.

## Results

### Diagnostic value of HULC and miR-512 in CIN

**Figure 1:**
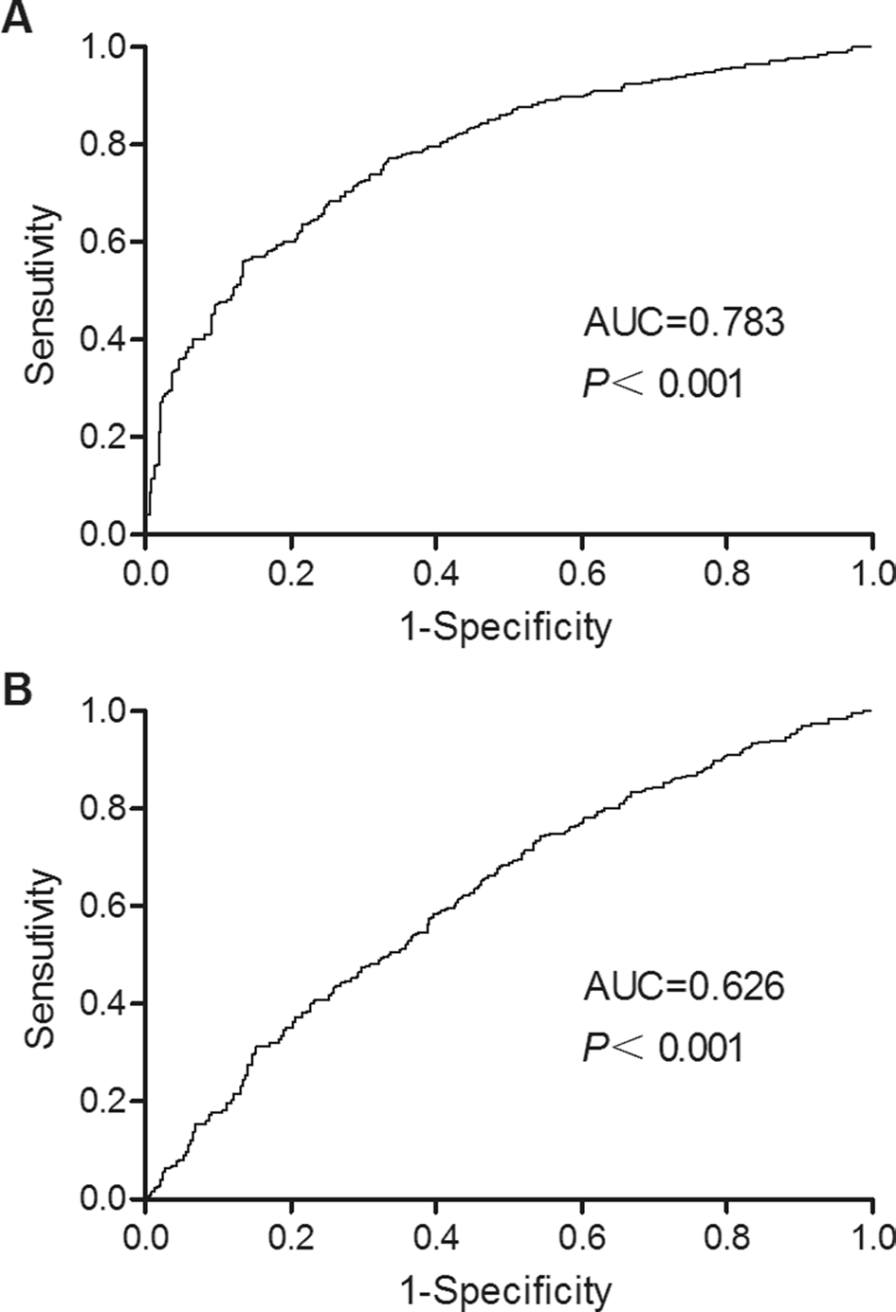
Diagnostic value of HULC and miR-512 in CIN. (**A**) ROC curve for the diagnosis of CIN by HULC expression. (**B**) ROC curve for the diagnosis of CIN by miR-512 expression.

In this study, we enrolled 320 patients with coronary heart disease. All patients were treated by coronary intervention, including 78 patients with CIN (CIN group) and 242 patients without CIN (non-CIN group). The clinicopathological data of all patients were listed in Table 1. A further analysis on the curves of receiver operating characteristics (ROC) showed that the AUC of HULC and miR-512 was 0.78 and 0.62, respectively (Fig. 1).

**Table 1:** Demographic and clinical parameters of the subjects enrolled in this study.

Characteristics	Control group (N = 242)	CIN group (N = 78)	*P* value
Sex-no (%)			0.8411
Female	169 (69.8)	55 (70.5)	
Male	73 (30.2)	23 (29.5)	
Age-years	65.6 ± 8.4	66.1 ± 7.9	0.6251
Diabetes duration-years	11.5 ± 4.8	12.4 ± 6.1	0.3169
Reasons for contrast-enhanced CT-no (%)			0.6654
Pulmonary embolism	48 (19.8)	15 (19.2)	
Dissecting ancurysm	20 (8.3)	6 (7.7)	
Liver abscess	98 (40.5)	30 (38.5)	
Angina pectoris admitted with suspected myocardial infarction	76 (31.4)	43 (34.6)	
BMI (kg/m^2^)-no (%)			0.6248
< 18.5 (underweight)	15 (6.2)	7 (8.9)	
18.5–24.9 (normal)	192 (79.3)	63 (80.7)	
25.0–29.9 (overweight)	23 (9.5)	8 (10.4)	
30.0–34.9 (class I obesity)	12 (5.0)	0	
35.0–39.9 (class II obesity)	0	0	
≥ 40.0 (class II obesity)	0	0	
Hypertension-no (%)	145 (59.9)	45 (57.7)	0.8221
Using ACE-I/ARB-no (%)	58/128	20/44	0.7518
Dosage of contrast (ml)	85.4 ± 7.2	83.8 ± 6.5	0.7152
Using diuretics-no (%)	114 (47.1)	35 (44.9)	0.3841
FBG (mmol/l)	7.9 ± 3.2	7.8 ± 3.6	0.9871
HbA1c (%)	7.3 ± 2.8	7.1 ± 3.4	0.6841

### MiR-512 is negatively correlated with HULC and PGE1

**Figure 2:**
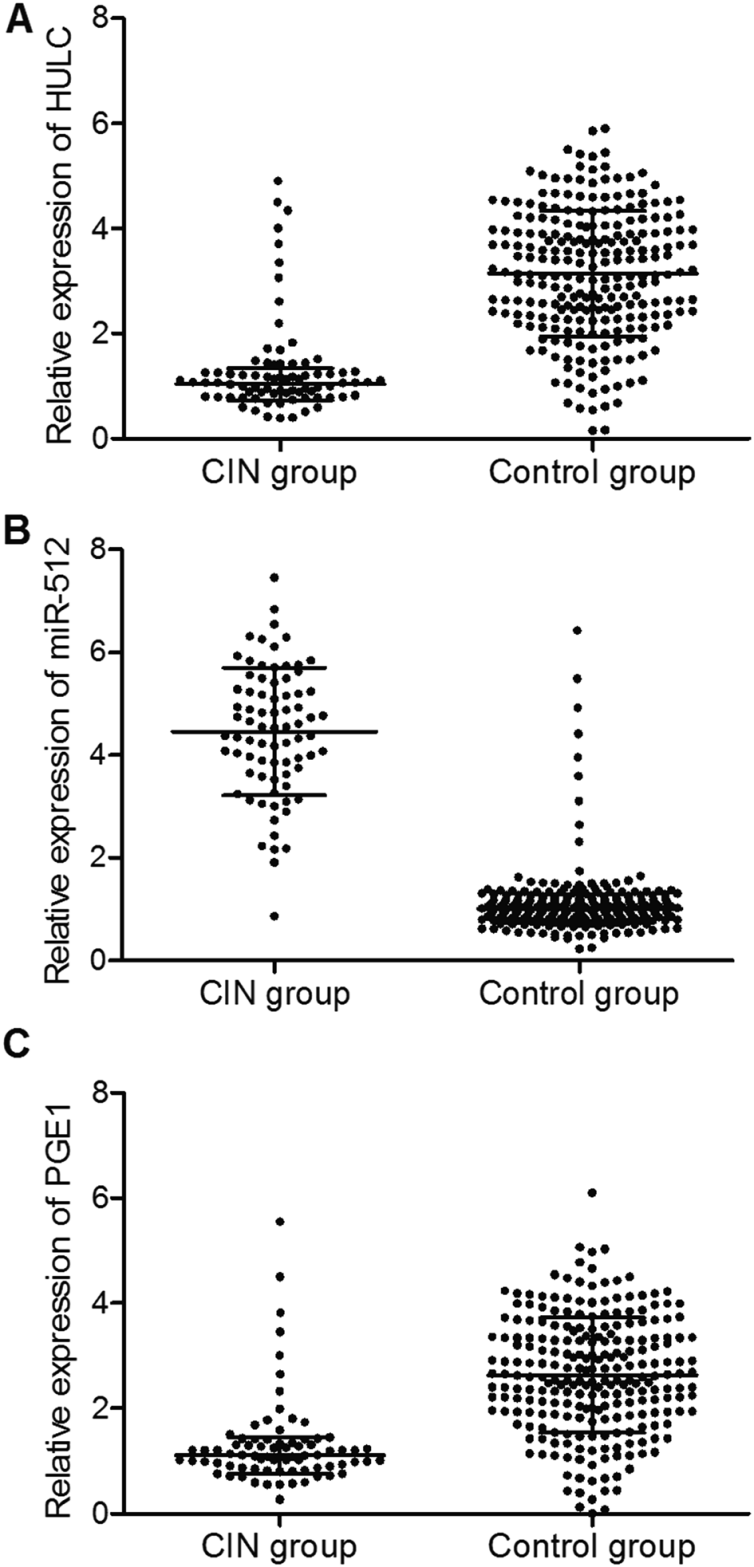
Expression levels of HULC, miR-512 and PGE1 in patients with/without CIN. (**A**) Serum levels of HULC in patients with/without CIN. (**B**) Serum levels of miR-512 in patients with/without CIN. (**C**) Serum levels of PGE1 in patients with/without CIN.

**Figure 3:**
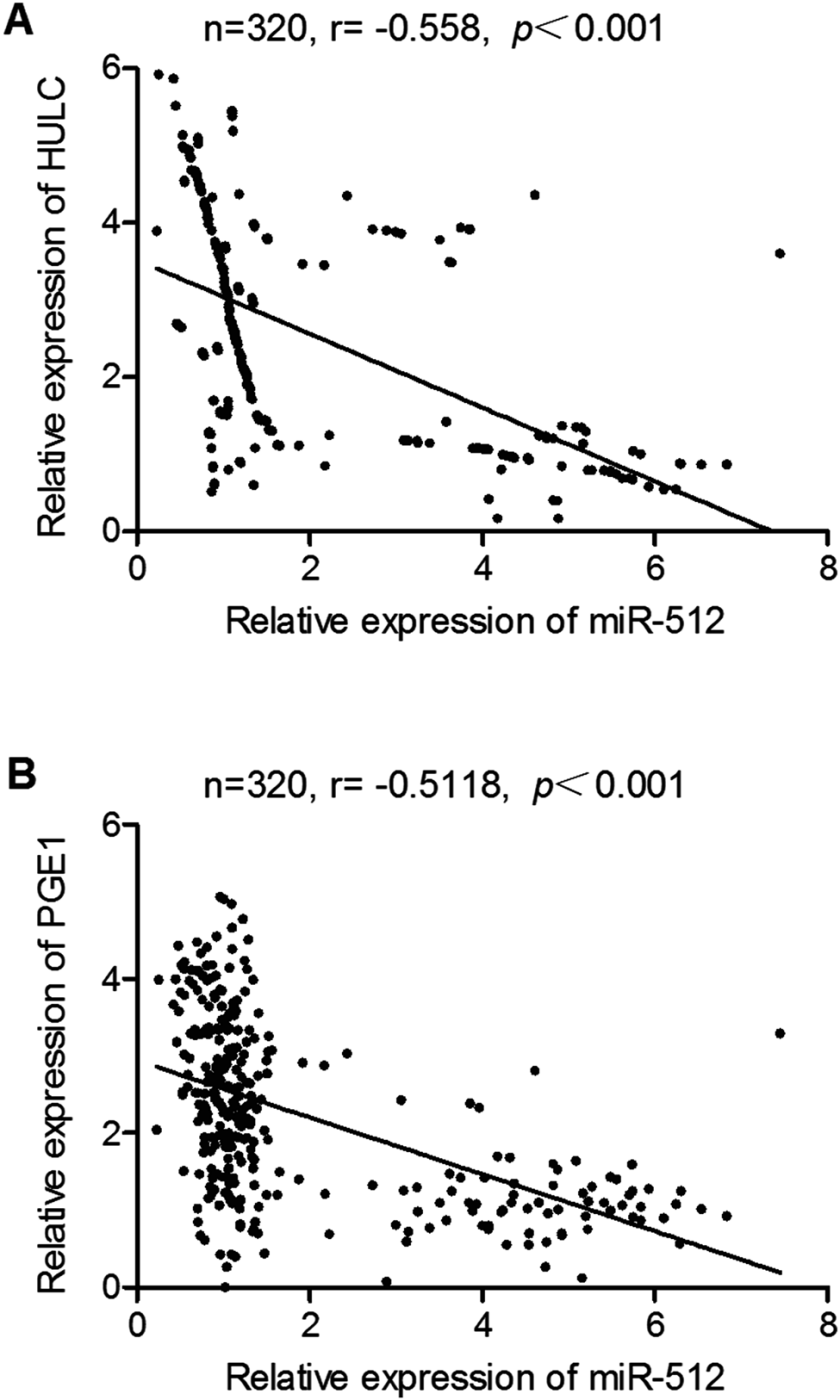
Relationship between miR-512 and HULC/PGE1. (**A**) Relationship between miR-512 and HULC. (**B**) Relationship between miR-512 and PGE1.

We then detected the expression of HULC, miR-512 and PGE1 in these two groups. As shown in Fig. 2, the patients with CIN showed higher expression of miR-512 (Fig. 2A) and lower expression of HULC (Fig. 2B) and PGE1 (Fig. 2C). We further analyzed the correlation between the expression of miR-512 and HULC (Fig. 3A) as well as the correlation between the expression of miR-512 and PGE1 (Fig. 3B). The results revealed a negative correlation between miR-512 and HULC/PGE1.

### Regulatory relationships between miR-512 and HULC/PTGS1

**Figure 4:**
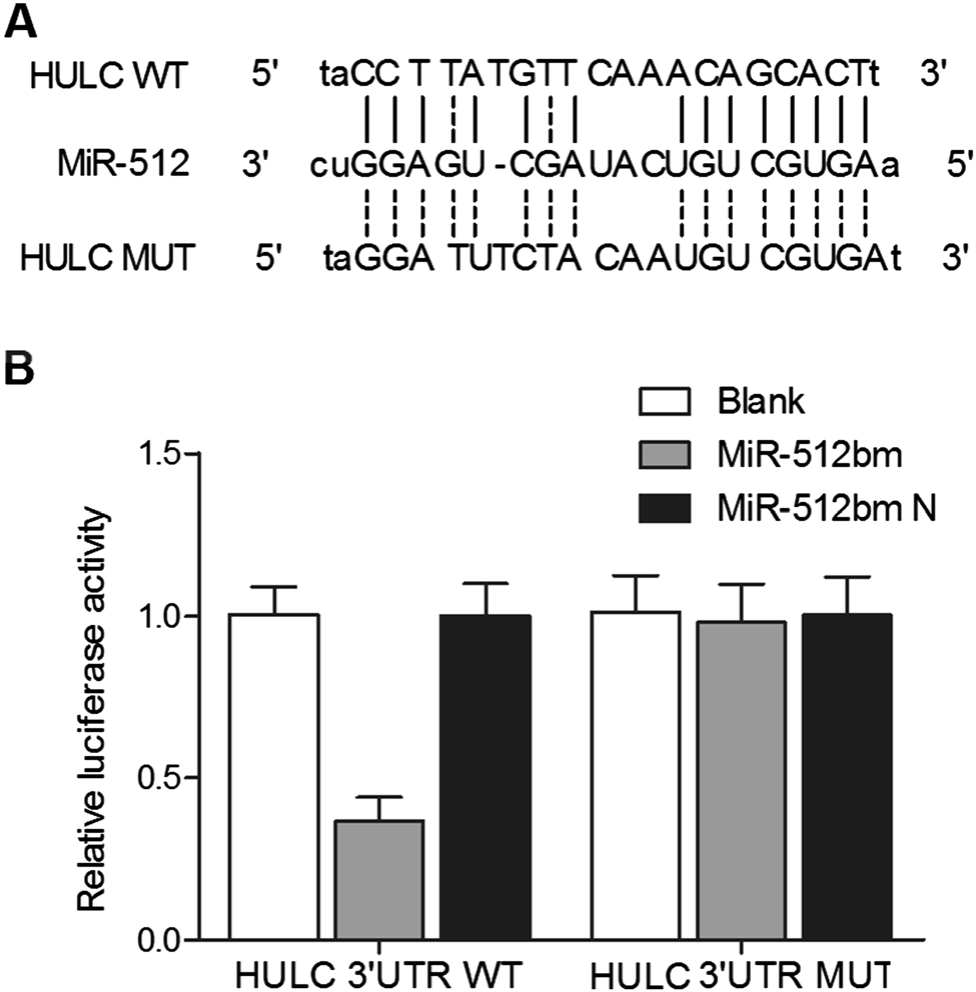
MiR-512 negatively regulated HULC expression in THP-1 cells. (**A**) Putative binding sites of miR-512 on the 3′-UTR of HULC (white sequences) were predicted by TargetScan. (**B**) MiR-512 down-regulated the luciferase activity of wild-type HULC 3′-UTR, but did not affect the luciferase activity of mutant HULC 3′-UTR (the white sequences were mutated).

**Figure 5:**
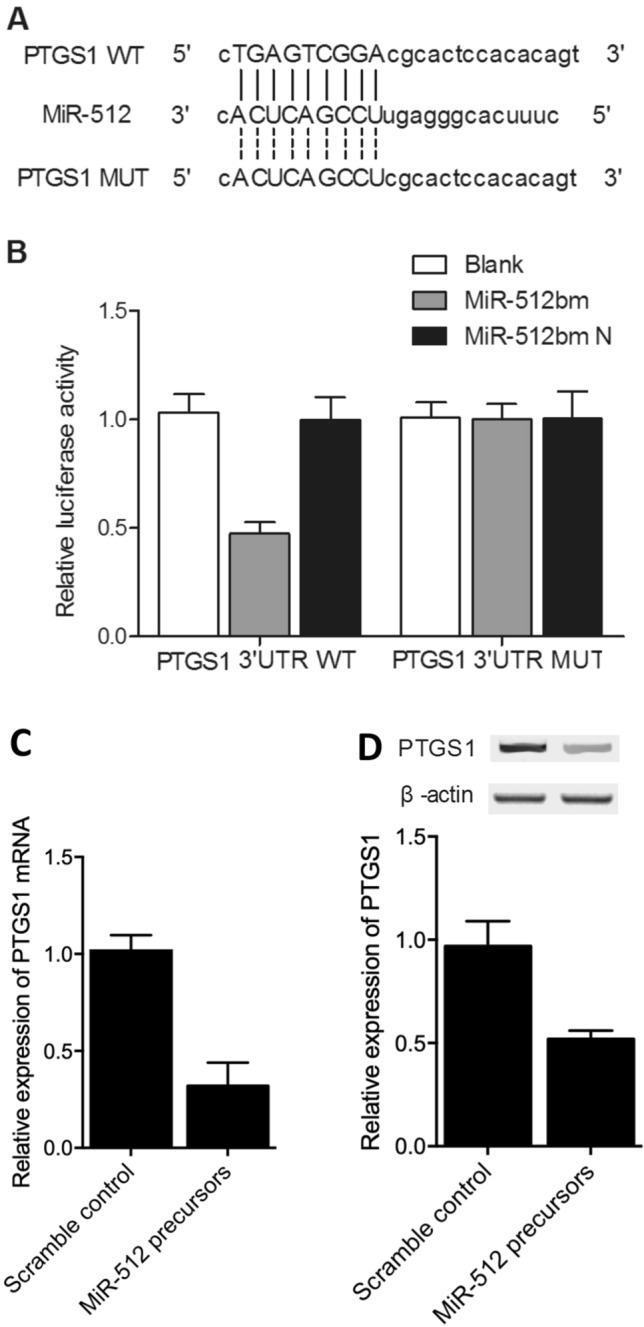
MiR-512 negatively regulated PTGS1 expression in THP-1 cells. (**A**) Putative binding sites of miR-512 on the 3′-UTR of PTGS1 (white sequences) were predicted by TargetScan. (**B**) MiR-512 down-regulated the luciferase activity of wild-type PTGS1 3′-UTR, but did not affect the luciferase activity of mutant PTGS1 3′-UTR (the white sequences were mutated). (**C**) MiR-512 precursors down-regulated the expression of PTGS1 mRNA. (**D**) MiR-512 precursors down-regulated the expression of PTGS1 protein.

TargetScan, Pictar-Vert, and Microrna.org were employed in this study to search for potential targets of miR-512 involved in CIN. HULC and PTGS1 were identified as potential targets of miR-512. Both the 3′-UTRs of HULC and PTGS1 carried a binding site for miR-512 (Figs. 4A, 5A), suggesting that HULC and PTGS1 mRNAs may act as direct targets of miR-512. To verify the role of HULC and PTGS1 mRNAs as targets of miR-512, we constructed vectors containing wild-type or mutant 3′-UTRs of HULC/PTGS1 (Figs. 4B, 5B). The wild-type and mutant vectors were then co-transfected into THP-1 cells with miR-512 or miR-512 NC. The transfection efficiency was normalized by the transfection with a Renilla reporter vector. As shown in Figs. 4B and 5B, miR-512 significantly decreased the relative luciferase activity of wild-type HULC and PTGS1 3′-UTRs (by more than 60%), whereas the reduction in the luciferase activity of mutant HULC and PTGS1 3′-UTRs was not as significant, suggesting that miR-512 could directly bind to the 3′-UTRs of HULC and PTGS1. Also, THP-1 cells were respectively transfected with miR-512 precursors or scramble control miRNAs. Accordingly, the expression of PTGS1 mRNA (Fig. 5C) and protein (Fig. 5D) were both down-regulated by the transfection of miR-512 precursors. Taken together, these findings indicated that HULC and PTGS1 are direct targets of miR-512.

### Regulatory relationships among HULC, miR-512, PTGS1 and PGE1

**Figure 6:**
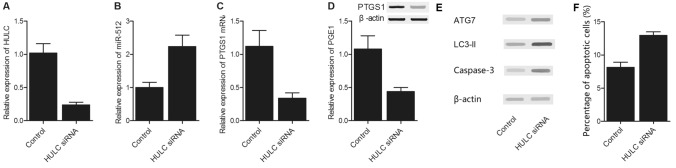
Regulatory relationships among HULC, miR-512, PTGS1 and PGE1 in THP-1 cells transfected with si-HULC. (**A**) Expression level of HULC in THP-1 cells transfected with si-HULC or control siRNA. (**B**) Expression level of miR-512 in THP-1 cells transfected with si-HULC or control siRNA. (**C**) Expression levels of PTGS1 mRNA in THP-1 cells transfected with si-HULC or control siRNA. (**D**) Expression level of PGE1 mRNA in THP-1 cells transfected with si-HULC or control siRNA. (**E**) Expression level of autophagy indicative marker proteins including ATG7, LC3-II and caspase-3 in THP-1 cells transfected with si-HULC or control siRNA. (**F**) Cell apoptosis of THP-1 cells transfected with si-HULC or control siRNA.

**Fig.7:**
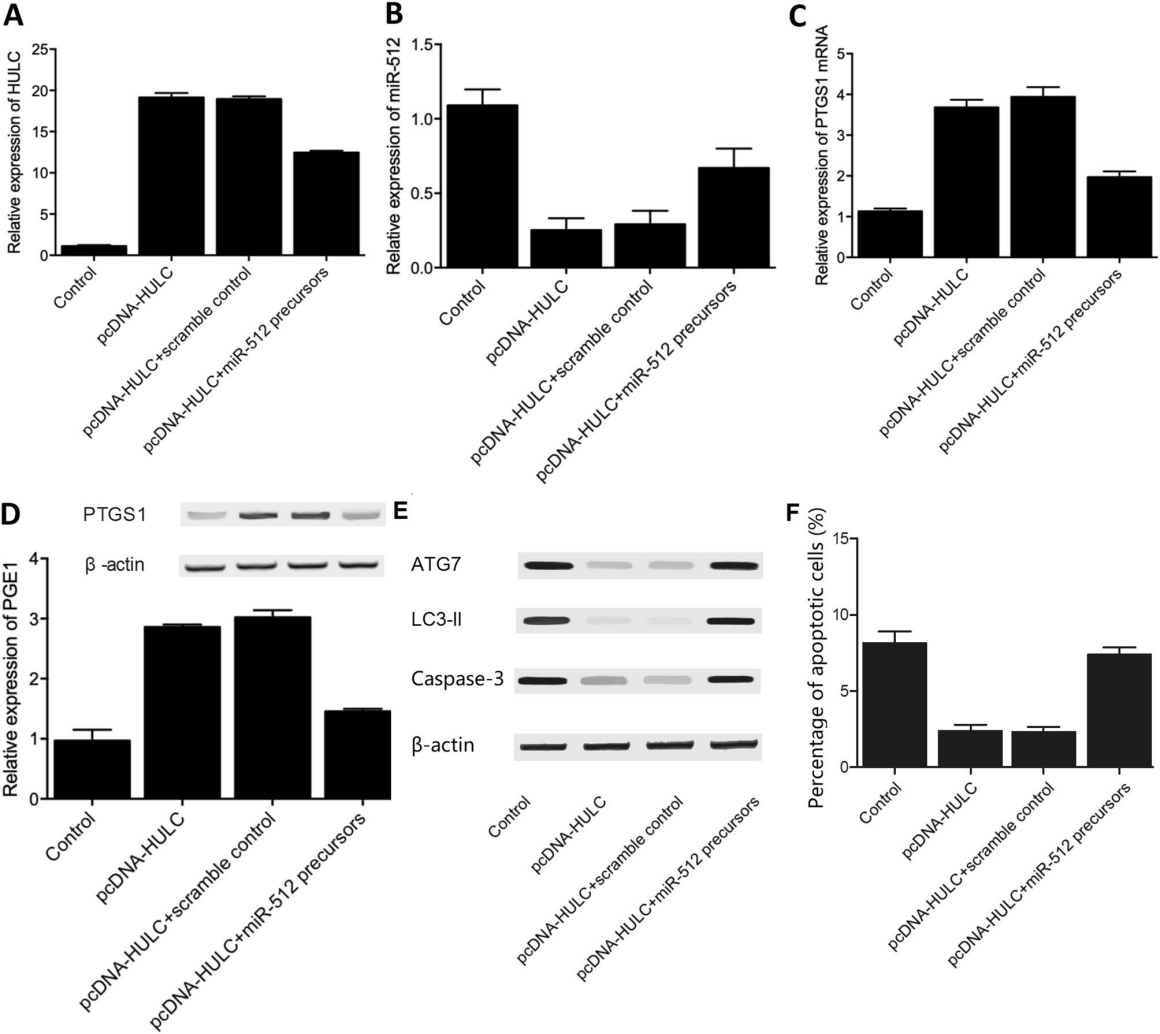
Regulatory relationships among HULC, miR-512, PTGS1 and PGE1 in THP-1 cells transfected with an empty vector, pcDNA-HULC, pcDNA-HULC + scramble control, or pcDNA-HULC + miR-512 precursors. (**A**) Expression level of HULC in THP-1 cells transfected with an empty vector, pcDNA-HULC, pcDNA-HULC + scramble control, or pcDNA-HULC + miR-512 precursors. (**B**) Expression level of miR-512 in THP-1 cells transfected with an empty vector, pcDNA-HULC, pcDNA-HULC + scramble control, or pcDNA-HULC + miR-512 precursors. (**C**) Expression levels of PTGS1 mRNA in THP-1 cells transfected with an empty vector, pcDNA-HULC, pcDNA-HULC + scramble control, or pcDNA-HULC + miR-512 precursors. (**D**) Expression level of PGE1 mRNA in THP-1 cells transfected with an empty vector, pcDNA-HULC, pcDNA-HULC + scramble control, or pcDNA-HULC + miR-512 precursors. (**E**) Expression level of autophagy indicative marker proteins including ATG7, LC3-II and caspase-3 in THP-1 cells transfected with an empty vector, pcDNA-HULC, pcDNA-HULC + scramble control, or pcDNA-HULC + miR-512 precursors. (**F**) Cell apoptosis of THP-1 cells transfected with an empty vector, pcDNA-HULC, pcDNA-HULC + scramble control, or pcDNA-HULC + miR-512 precursors.

To further evaluate the regulatory relationships among HULC, miR-512, PTGS1 and PGE1, we transfected THP-1 cells with si-HULC or pcDNA-HULC. As shown in Fig. 6, upon the successful transfection of si-HULC (Fig. 6A), the level of miR-512 (Fig. 6B) was increased in the THP-1 cells, along with decreased levels of PTGS1 mRNA (Fig. 6C) and PGE1 protein (Fig. 6D). Moreover, by observing the effect of si-HULC on autophagy indicative marker proteins (Fig. 6E), a significant increase of ATG7, LC3-II and caspase-3 upon the transfection of si-HULC in THP-1 cells was presented by the Western blot results, indicating the promoted level of autophagy. And the according apoptosis of THP-1 cells was also aggravated by the knockdown of HULC (Fig. 6F). On the contrary, in the THP-1 cells transfected with pcDNA-HULC or co-transfection of pcDNA-HULC and scramble controls (Fig. 7A), the level of miR-512 (Fig. 7B), as well as autophagy indicative marker proteins (Fig. 7E), was decreased along with increased levels of PTGS1 mRNA (Fig. 7C) and PGE1 protein (Fig. 7D), while the co-transfection of pcDNA-HULC and miR-512 precursors in THP-1 cells (Fig. 7A) partly reverted the increased PTGS1 mRNA (Fig. 7C) and PGE1 mRNA (Fig. 7D) levels. And the cell apoptosis was also suppressed by the presence of pcDNA-HULC, which was partly restored by the co-transfection of miR-512 precursors (Fig. 7F). These results indicated the presence of a negative relationship between HULC and miR-512, whereas a positive relationship was present between HULC and PTGS1/PGE1.

## Discussion

CIN is a frequently diagnosed complication in diabetes patients undergoing contrast-enhanced CT^20^. Due to its serious consequences, such as extended hospitalization and increased mortality/mortality associated with the increased risks of renal dysfunction, CIN has become a research hotspot in recent years^21^. In this study, we enrolled 320 patients with coronary heart disease. All patients have undergone coronary intervention, including 78 patients with CIN and 242 patients without CIN. The patients with CIN showed lower levels of HULC/PGE1 expression and a higher level of miR-512 expression. In addition, a negative correlation between miR-512 and HULC/PGE1 was found.

PGE1 is a naturally occurring prostaglandin that can exert multiple effects, such as inhibiting the aggregation and activation of thrombocytes and neutrophils, protecting endothelial cells, and triggering vasodilation^22^. Moreover, PGE1 has been widely used to treat ischemic heart diseases. Clinical studies have shown the potential of using PGE1 to alleviate ischemia–reperfusion injury and to improve myocardial microcirculations^23^. Cyclooxygenases, also termed as prostaglandin H synthases, have two isoforms, i.e., COX-1/COX-2. Recently, COX-1 has emerged as an important player in the neuroinflammation of the CNS^24^. In aging rats, the brain expression of COX-1 and TXB2 is increased to render brain more susceptible to neurodegeneration and inflammation. Indeed, COX-1 is implicated in many inflammatory processes by increasing the level of PG^25^. In this study, HULC and PTGS1 were identified as potential targets of miR-512. The validity of such regulatory relationships was confirmed by co-transfecting THP-1 cells with wild-type/mutant 3′-UTRs of HULC/PTGS1 and miR-512/NC. The results revealed that miR-512 could directly bind to the 3′-UTRs of HULC and PTGS1. Meanwhile, after THP-1 cells were transfected with si-HULC or pcDNA-HULC, the results indicated the presence of a negative relationship between HULC and miR-512, as well as the presence of a positive relationship between HULC and PTGS1/PGE1.

COX-2, an inducible type of COX, acts as an important mediator of cancer metastasis. In fact, the overexpression of COX-2 is frequently seen in advanced lung cancer with poor prognosis^26^. The elevation of COX-2 expression in tumor tissues also promotes the expression of prostaglandin E2 (PGE2), a metabolite of COX-2. PGE2 can act as a ligand for G protein-coupled receptors to stimulate the activation of ERK1/2 and PI3K/Akt pathways, which in turn increase the invasiveness of cancer cells and induce tumorigenesis^27^. Recently, it was shown that, by reducing the expression of protein phosphatase 2A (PP2A), COX-2/PGE2 can induce the expression of migration inducing gene-7 (MIG-7) to activate the PI3K/Akt/GSK-3β pathway and subsequently promote lung cancer metastasis^28^. Many strategies, including the use of statins, antioxidants, and hydration agents, have been adopted to prevent CIN^5^. As an exogenous form of PGE1, Alprostadil can reduce radiocontrast-associated nephrotoxicity^29^. Sketch also showed that PGE1 apparently decreased the level of serum creatinine in patients of renal insufficiency, suggesting that vasodilators may reduce the risk of CIN^30^. COX-2 can be found in different parts of the kidney. By promoting the synthesis of prostaglandins, COX-2 is also implicated in the continuous synthesis of prostanoids by the kidney to control renal functions. The most important function of COX-2 is to convert arachidonic acids to PGD2, TXA2, PGI2, and PGE2 via specific synthases^31^. As a key product derived from the metabolism of arachidonic acids, PGE2 plays important roles in regulating inflammatory responses and pain^32^. An isoform of PGES, mPGES-1, is highly expressed in the lungs, spleen and macrophages upon proinflammatory stimulation^33,34^. Furthermore, COX-2 also exerts an important effect on cisplatin-induced renal injury^35^. For example, three days of treatment with 20 mg/kg of cisplatin significantly upregulated the levels of COX-2, mPGES-1 and PGE2 in the kidney, although the treatment with cisplatin showed no effect on the expression of COX-2, cytosolic PGES, and mPGES-2. Nevertheless, mPGES-1 knockout mice are resistant to cisplatin-induced renal injury by suppressing the activity of inflammatory cytokines. The above results demonstrated that the renal activation of the COX-2/mPGES-1 pathway may regulate cisplatin-induced nephrotoxicity. Similarly, another study showed that a five-day administration of 13 mg/kg of cisplatin in mice increased the level of COX-2 in renal interstitial cells, while the concomitant administration of a COX-2 selective inhibitor and cisplatin alleviated cisplatin-induced renal injury by reducing the levels of oxidative stress and inflammatory responses^36^.

There are limitations in this study: firstly, the THP-1 cells used in this study is a cell line of peripheral blood monocytes rather than a renal cell line and more functional analysis using cell lines of different origins especially renal origins are needed to confirm the conclusion of the present study. Secondly, the sample size of this study is relatively small and the sampled were not renal tissuses. Further study with large scale samples especially with kidney samples are warranted. Lastly, more sophisticated and comprehensive functional analysis to further explore the underlying molecular mechanism is also needed to identify the complexed regulatory signaling pathways and networks involved in the pathogenesis of CIN.

## Conclusion

In summary, the findings of this study demonstrated that the deregulation of lncRNA-HULC and miR-512 might be involved in the pathogenesis of CIN. Furthermore, we also found that PTGS1, a key enzyme of PGE1, acted as a direct target of miR-512, and the dysregulation of PTGS1/PGE1 could be the downstream effectors of HULC and miR-512 involved in the pathogenesis of CIN.
